# Acute Bacterial Meningitis and Systemic Abscesses due to* Streptococcus dysgalactiae* subsp.* equisimilis* Infection

**DOI:** 10.1155/2017/8645859

**Published:** 2017-04-23

**Authors:** M. Jourani, T. Duprez, V. Roelants, H. Rodriguez-Villalobos, P. Hantson

**Affiliations:** ^1^Department of Intensive Care, Cliniques St-Luc, Université Catholique de Louvain, Brussels, Belgium; ^2^Department of Radiology, Neuroradiology Division, Cliniques St-Luc, Université Catholique de Louvain, Brussels, Belgium; ^3^Department of Nuclear Medicine, Cliniques St-Luc, Université Catholique de Louvain, Brussels, Belgium; ^4^Laboratory of Microbiology, Cliniques St-Luc, Université Catholique de Louvain, Brussels, Belgium

## Abstract

Disseminated abscesses due to group G *β*-hemolytic* Streptococcus dysgalactiae* were observed in a 57-year-old cirrhotic patient with the skin being the putative way of entry for the pathogen.* S. dysgalactiae* is a rare agent in human infections responsible for acute pyogenic meningitis. The mortality rate associated with* S. dysgalactiae* bacteraemia and meningitis may be as high as 50%, particularly in the presence of endocarditis or brain abscesses. In our patient, main sites of infections were meningitis and ventriculitis, spondylodiscitis, septic arthritis, and soft-tissue infections. In contrast, no endocarditis was evidenced. Cirrhosis-related immune suppression was considered as a pathophysiological cofactor for the condition. Fortunately, clinical status improved after long-term (3 months) antimicrobial therapy.

## 1. Introduction

Meningitis due to* Streptococcus dysgalactiae* has been reported in a very restricted number of adult or pediatric cases, usually with a mild clinical course [1–7]. It is usually thought that streptococcal meningitis is acquired via transient colonization of the nasopharynx, followed by bacteraemia and subsequent invasion of the central nervous system (CNS). Although invasive infection (including shock, renal and liver involvement, and soft-tissue necrosis, but not meningitis) caused by Japanese strains of* S. dysgalactiae* has been reported*, S. dysgalactiae* usually carries a low pathogenic potential [8]. We describe a case of* S. dysgalactiae* bacteraemia complicated by meningitis and disseminated abscesses in a cirrhotic patient with favorable outcome.

## 2. Case Report

A 57-year-old man was found lying on the floor of his room. He had a medical history of heavy chronic ethanol consumption (more than 10 units/day for 30 years) but never underwent hepatic investigations. More than 10 years ago, he had had prosthetic replacement of the right knee. He had also received dental care more than two months before symptoms onset. A few weeks before hospital admission, he had a traumatic fracture of the right calcaneus treated by plaster cast immobilization.

At admission to Emergency Department, he presented with a left-sided hemiparesis, dysarthria, and confusion. Glasgow coma scale (GCS) score was calculated at 13/15 (E4, V3, M6). There was a mild meningeal irritation. The body temperature was 36.7°C, and blood pressure was 125/66 mmHg. Examination of the pharynx was unremarkable. After cast removal, a cellulitis of the right leg was diagnosed together with a thrombosis of the right posterior tibial and peroneal veins. Antimicrobial treatment was immediately initiated with intravenous amoxicillin-clavulanic acid (1 g every 6 h). Both knees were swollen, erythematous, and painful, as well as both elbows, and multifocal septic arthritis was suspected.

Laboratory investigations showed a C-reactive protein (CRP) at 333 mg/dL (normal value < 5 mg/L) and white blood cell count of 19850 cells/mm^3^ (95.1% granulocytes). Other standard laboratory findings were within normal range, except for an international normalized ratio (INR) at 2.37, and serum CK level at 1569 IU/l (normal value < 200). Microscopic hematuria and mild proteinuria were noted at urine analysis. The brain computed tomography (CT) scan did not reveal any specific lesion as well as fundoscopic examination. A lumbar puncture was performed and cerebrospinal fluid (CSF) examination showed white blood cells count of 1127 cells/mm^3^ (64% granulocytes), glucose not detectable, and protein of 1848 mg/dL. The CSF Gram-staining revealed the presence of rare white blood cells but no organism. Two sets of blood cultures were positive for* S. dysgalactiae*, while CSF culture remained sterile after seven days of incubation including enriched culture media.* S. dysgalactiae* was not isolated from the skin, nose, and throat swaps. Blood cultures became sterile 24 hours after the start of antimicrobial therapy. The universal PCR 16S rRNA gene sequencing (qPCR16S) using SQ1-S (50-AgAgTTTgATCCTggCTCAg-30), SQ1-AS (50-AAggAGGTgATCCARCCgCA-30), SQ2-AS (5′-gggTTgCgCTCgTTG-30), and SQ3-AS 50-TCTACgCATTTCACCgCTAC-3 primers (96GA3730 XL, Applied Biosystem, USA) applied directly to CSF samples was negative. There was no evidence of endocarditis at echocardiography.

Surgical treatment was required for the right prosthetic knee and culture of purulent material yielded* S. dysgalactiae* subsp.* equisimilis*. Aerobic and anaerobic cultures of purulent material from the left knee and both elbows remained sterile after seven days of incubation.

The search for metastatic foci included total body computed tomography (CT) scan examination, spine and brain magnetic resonance imaging (MRI), and total body fluorodeoxyglucose positron emission tomography/computed tomography (FDG PET-CT).

**Figure 1:**
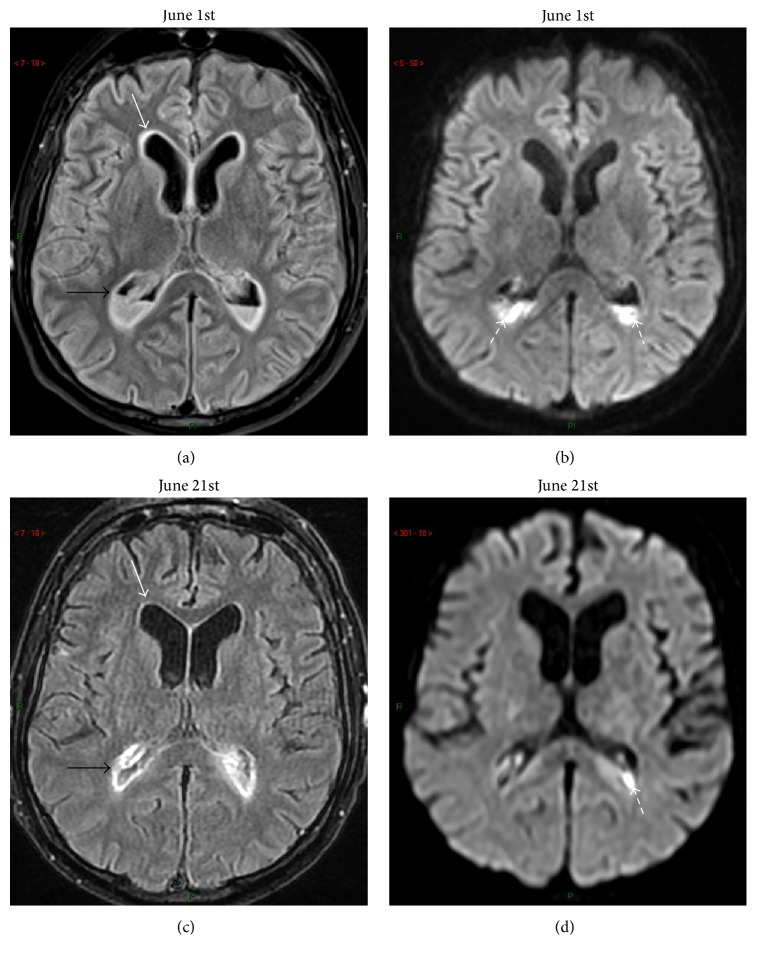
(a-b) Admission brain MRI; (c-d) follow-up brain MRI three weeks later. (a) Contrast-enhanced (CE) axial transverse fluid-attenuated inversion-recovery (FLAIR) view at admission showing diffuse abnormal enhancement of ependyma (white arrow) and bilateral fluid-fluid level at trigonal areas of lateral ventricles (black arrow). (b) Admission diffusion-weighted (DW) axial transverse view at admission at similar slice location as in (a) showing high signal intensity within declive sediment of the fluid-fluid level featuring pus with free water diffusivity restriction (dotted arrows). (c) CE FLAIR view in similar slice location as in (a) three weeks later showing complete subsidence of ependymal contrast-enhancement (white arrow) and disappearance of the fluid-fluid levels (black arrow). Only persistent enhanced brightness of ependymal lining was seen. (d) DW image in similar slice location as in (b) showing only minimal purulent residue within left ventricular trigone (dotted arrow).

Brain magnetic resonance imaging (MRI) was performed on admission and three weeks later and was consistent with acute ventriculitis without evidence of brain abscesses (Figure 1).

**Figure 2:**
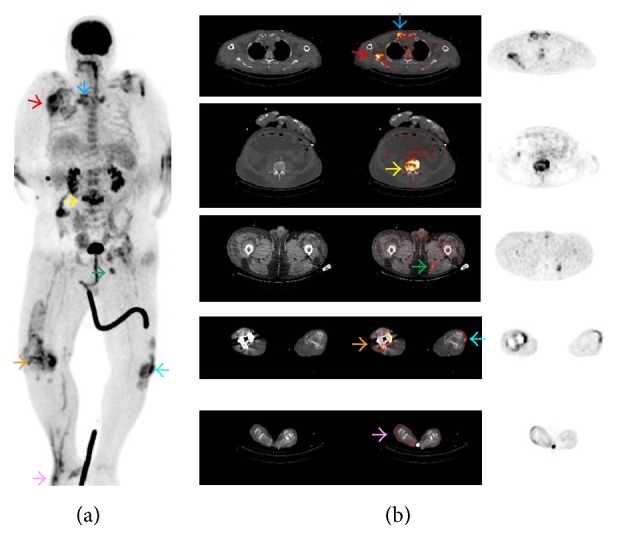
(a) MIP (maximum intensity projection) of FDG PET showing multiple areas of hypermetabolic activity corresponding to disseminated foci of infection and (b) transaxial views of CT, fused FDG PET-CT and FDG PET images representative of several foci of infection disseminated in joints, soft tissues, and lumbar column (arrows).

FDG PET-CT revealed multiple areas of hypermetabolic activity in joints, soft tissues, and spine corresponding to disseminated infection (Figure 2). Mainly, L2-L3 intervertebral space, sternoclavicular joints, knees, left hip, elbows, left shoulder, left deltoid muscle, right subscapularis muscle, left obturator internus muscle, and gluteal muscles were involved. No abnormal metabolic activity was observed in the cardiac area and no abnormal FDG uptake leading to cancer suspicion was observed. No abnormal metabolic activity was observed in the cardiac area and no abnormal FDG uptake leading to cancer suspicion was observed.

In addition, spinal MRI demonstrated diffuse and severe intrathecal inflammatory changes together with a L2-L3 spondylodiscitis with only moderate involvement of adjacent epidural spaces (not shown).

On day 3, antimicrobial therapy was switched to intravenous ceftriaxone (2 g/every 12 h) and ampicillin (1 g/every 6 h) and thereafter to rifampicin 600 mg/day. Hepatic fibrosis stage F4 was diagnosed by ultrasound elastography (FibroScan®). At 3-month follow-up, the patient had a complete functional recovery normalized inflammatory markers, and antimicrobial therapy was thereafter discontinued.

## 3. Discussion

*Streptococcus dysgalactiae* subsp.* equisimilis* is a human-pathogenic betahaemolytic pyogenic* Streptococcus* harboring the Lancefield group G, C, or L antigens. Pathogenic strains harbor virulence genes similar to virulence genes to* S. pyogenes* and have been associated with upper respiratory tract infection (pharyngitis, mainly in children), skin and soft-tissue infections, and invasive infections. While* Streptococcus dysgalactiae* subsp.* equisimilis* may belong to the commensal oropharyngeal flora, invasive infections may be caused by person-to-person transmission [9]. An increasing number of patients with severe invasive* S. dysgalactiae* infection have been observed in Japan during the early 2000s [8]. The clinical course included shock and a varying number of patients with renal impairment, disseminated intravascular coagulation, liver involvement, adult respiratory distress syndrome (ARDS), generalized erythematous macular rash, and soft-tissue necrosis. The strains did not seem to expand clonally but they were carrying specific genes-related virulence (s*cpA, ska, slo, *and* sag *genes). People developing a severe infection with this organism were likely to suffer cancer in 21–65% of cases or to be immunocompromised by diabetes mellitus, cirrhosis, or alcohol abuse [10–14].

Acute bacterial meningitis could result either by hematogenous spreading to the CNS or from a local extension to the subarachnoid spaces [15]. It seems important to exclude the presence of cerebral abscesses when meningitis is diagnosed and to look for endocarditis, which is present in most cases of group G* Streptococcus* meningitis. The mortality rate associated with group G* Streptococcus* meningitis is not precisely established but could be as high as 50%. In the hereby reported case, endocarditis could be ruled out by repeatedly negative cardiac echography. In the case reported here, CSF culture and universal 16S rRNA PCR were negative. Because of low sensitivity and false positive and negative results, the qPCR16S test applied directly in samples should be interpreted with caution and needs the use in parallel of microbiological cultures. However, the final diagnosis of bacterial meningitis which is generally based on CSF microbiological cultures can be inconclusive in 20% of cases of patients with community bacterial acquired meningitis, rising to less than 50% of cases if patients have received prior antimicrobial therapy. In the present observation, hematogenous origin of meningeal infection was considered more likely than focal contamination from epidural infection.
